# Capturing the signal

**DOI:** 10.7554/eLife.09315

**Published:** 2015-07-09

**Authors:** Jee-Young Mock, William M Clemons

**Affiliations:** Division of Chemistry and Chemical Engineering, California Institute of Technology, Pasadena, United States; Division of Chemistry and Chemical Engineering, California Institute of Technology, Pasadena, United Statesclemons@caltech.edu

**Keywords:** rabbit, protein targeting, cryo-EM, membrane protein biogenesis, signal recognition particle, other

## Abstract

High-resolution structures provide new insights into how an RNA-protein complex recognizes the signal that targets membrane proteins to the endoplasmic reticulum before they aggregate.

**Related research article** Voorhees RM, Hegde RS. 2015. Structures of the scanning and engaged states of the mammalian SRP-ribosome complex. *eLife* **4**:e07975. doi: 10.7554/eLife.07975

**Image** Close-up view of a signal recognition particle (green) as it waits at the exit tunnel of a ribosome ready to engage with the newly forming protein (blue)

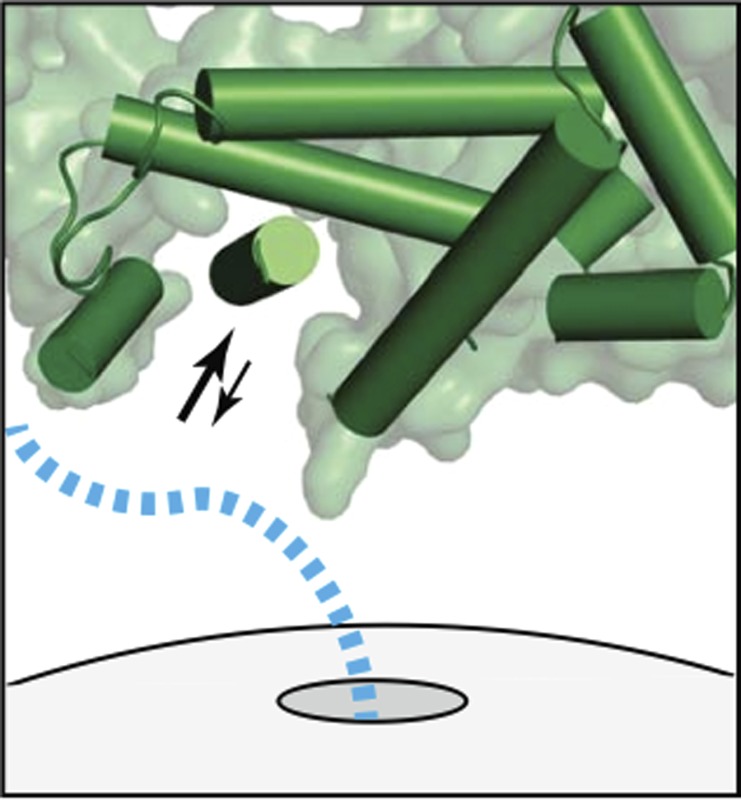

About a quarter of eukaryotic genes encode membrane proteins, many of which perform activities that are essential for cells to survive (Wallin and von Heijne, 1998). Nevertheless, the production of these proteins poses a problem for the cell. Like all proteins, membrane proteins are built by ribosomes within the cytosol, which is an aqueous and crowded environment (Ellis, 2001). However, the transmembrane domains of membrane proteins are hydrophobic (or ‘water-hating’) and are therefore prone to aggregating in such an environment. This means that they must be promptly sent to their destination. For most eukaryotic membrane proteins, this destination is the membrane of the endoplasmic reticulum.

In 1971, Blobel and Sabitini proposed a possible solution to this problem: the proteins that are destined for the endoplasmic reticulum (i.e., membrane proteins and secreted proteins) contain a signal peptide that is recognized by the ribosome. Subsequent studies confirmed the existence of this signal peptide (Blobel and Dobberstein, 1975), and the signal recognition particle (SRP), which actively targets the proteins to the endoplasmic reticulum, was also eventually identified (Walter and Blobel, 1981a).

The SRP is thought to bind to and protect the signal peptide (which is typically also the first transmembrane domain for membrane proteins) when it emerges from the ribosome. This halts the building of the protein until the entire complex (including the ribosome, the SRP, the messenger RNA and the newly forming protein) arrives at the membrane of the endoplasmic reticulum. Although this process has been extensively characterized since its discovery, several important details of the molecular interplay between the mammalian ribosome and the SRP remain elusive. Our current structural understanding of the SRP-ribosome complex in eukaryotes has been limited to a 7.4-Å reconstruction of an artificial complex formed by a plant ribosome and a mammalian SRP (Halic et al., 2006). It also remains unclear how and when the SRP is recruited to the ribosome. Now, in *eLife*, Rebecca Voorhees and Ramanujan Hegde from the MRC Laboratory of Molecular Biology report new insights into how a mammalian SRP interacts with the mammalian ribosome to recognize the signal peptide (Voorhees and Hegde, 2015).

First, Voorhees and Hegde examined precisely when the SRP binds by using shortened versions of messenger RNA molecules to trap the SRP-ribosome complex at distinct points in the translation of a membrane protein. A ‘scanning’ state was captured while the first transmembrane domain was inside the ribosome; and an ‘engaged’ state was captured when the SRP gained access to the transmembrane domain after it had emerged from the ribosome (Figure 1). Voorhees and Hegde then used electron cryo-microscopy (cryo-EM) to visualize the scanning and engaged complexes to resolutions of 3.8 Å and 3.9 Å, respectively: these are the highest resolution reconstructions of the SRP-ribosome complex to date. These data suggest that for membrane proteins, the SRP is recruited to the ribosome before the signal peptide emerges, as first proposed 20 years ago (Ogg and Walter, 1995). Importantly, this ‘anticipatory binding’ seems to be specific for the signal peptides of membrane proteins, which are slightly longer than the signal peptides of secreted proteins.

**Figure 1.**
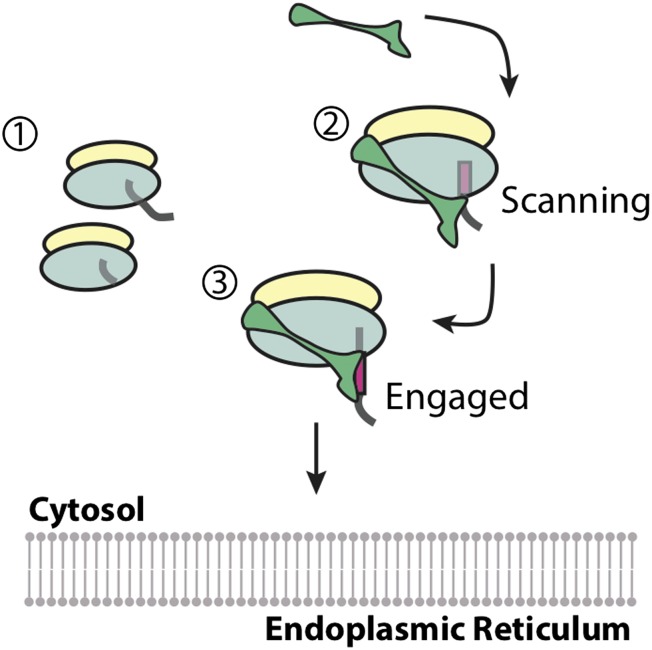
Recruitment of the signal recognition particle (SRP) to a ribosome translating a membrane protein. (1) Ribosomes that are not building proteins destined for the endoplasmic reticulum do not recruit the SRP (shown in green). (2) Membrane proteins have hydrophobic segments (called transmembrane domains) that are eventually inserted into the membrane. Exposing these transmembrane domains in the aqueous cytosol can be detrimental to the cell. The first transmembrane domain acts as a ‘signal peptide’ (shown in magenta), and flags the ribosome and the newly forming protein chain for delivery to the membrane of the endoplasmic reticulum. Voorhees and Hegde have confirmed that the SRP binds the ribosome while this signal peptide is still inside the ribosome ensuring that the transmembrane domain is never exposed to the aqueous environment. This is referred to as the ‘scanning’ state. (3) When the signal peptide emerges from the ribosome it becomes accessible for binding to the SRP. The SRP undergoes a conformational change in order to accommodate the transmembrane domain, and protect it from the cytosol. This is called the ‘engaged’ state, and the entire complex is then delivered to the membrane of the endoplasmic reticulum.

These results raise a perplexing question. The classical model suggests that SRP binding would lock the ribosome into a conformation that is incompatible with the binding of other molecules called translational factors, which are needed for translation of the messenger RNA to occur (Halic et al., 2004). So how does the SRP remain bound to the ribosome before and after the emergence of the signal peptide, during which time the protein is still being translated? It should be noted that the artificial plant ribosome-mammalian SRP complex arrests translation more strongly than the naturally occurring complex (Walter and Blobel, 1981b; Wolin and Walter, 1989). Intriguingly, Voorhees and Hegde observe a stable three-part complex between the SRP, the ribosome and a translational factor. This lends support to the idea that SRP binding and translation are not mutually exclusive events under physiological conditions.

The new high-resolution structures also reveal that the hitherto unresolved portion of the SRP subunit that binds to the signal peptide contains two additional helices. These helices have at least two distinct roles. First, they occupy the hydrophobic groove of the substrate-binding domain of the SRP before it engages with the signal peptide. Second, they provide a protective lid for the signal peptide when it binds to the SRP. This finding sheds light on how the part of the SRP that binds to the signal peptide remains protected from the aqueous cytosol, which was unclear from previous structural studies (Keenan et al., 1998; Halic et al., 2004).

Since the first low-resolution cryo-EM reconstruction of the eukaryotic ribosome almost 20 years ago (Verschoor et al., 1996), rapid advances in data collection and processing have revolutionized the field, with the bacterial ribosome now resolved up to 2.9 Å (Fischer et al., 2015). Voorhees and Hegde combine this powerful tool with elegant biochemical methods to reveal how the SRP scans and captures the signal peptide. The data presented here not only challenge and refine the previous model of SRP-mediated targeting, they also motivate more questions. Namely, the lack of drastic conformational changes in the ribosome makes it clear that the signal peptide must trigger a more subtle change. What is the nature of this change, and how is it recognized by the SRP? The exciting combination of cryo-EM and biochemistry will help further refine the model for capturing the signal in the near future.
